# Work at high altitude and non-fatal cardiovascular disease associated with unfitness to work: Prospective cohort observation

**DOI:** 10.1371/journal.pone.0306046

**Published:** 2024-07-08

**Authors:** Denis Vinnikov, Akylbek Saktapov, Zhanna Romanova, Aliya Ualiyeva, Viktor Krasotski

**Affiliations:** 1 Occupational Health Risks Lab, Peoples’ Friendship University of Russia (RUDN University), Moscow, Russian Federation; 2 Environmental Health Science Lab, al-Farabi Kazakh National University, Almaty, Kazakhstan; 3 Department of Epidemiology, Biostatistics and Evidence-Based Medicine, al-Farabi Kazakh National University, Almaty, Kazakhstan; 4 Medical Department, Kumtor Gold Company, Bishkek, Kyrgyzstan; Universidad de Las Americas, Quito-Ecuador, ECUADOR

## Abstract

**Introduction:**

Mining at high altitude exposes workers to hypoxic environment and cold climate in addition to conventional hazards in mining, but very little is known on how to define fitness to work in prospective candidates with pre-existing conditions. The aim of the current study was to define the incidence of cardiovascular diseases leading to unfitness to work as well as their predictors in a prospective observation.

**Methods:**

A total of 569 prospective employees (median age 34 (interquartile range (IQR) 28;40) years, 95% men 85% mid-altitude residents) for a high-altitude gold mine in Kyrgyzstan operating at 3800–4500 meters above sea level were screened at pre-employment in 2009–2012 and followed by January 2022. Cox regression was used to quantify the association of baseline demographics and physiological variables with newly diagnosed cardiovascular diseases (CVD) leading to unfitness to work, expressed as hazard ratios (HRs) with 95% confidence intervals (CI).

**Results:**

With 5190 person-years of observation, 155 (27%) workers have left work, of whom 23 had a newly identified CVD leading to unfitness to work (cumulative incidence 4%) with no difference between drivers and other occupations, despite greater blood pressure and body mass index (BMI) in the former at baseline. Age (HR 1.13 (95% CI 1.06;1.22) and BMI (HR 1.18 (95% CI 1.04;1.34)) were associated with a greater chance of having CVD, adjusted for lung function, baseline diagnoses, year of employment and baseline blood pressure. Narrowing the analysis to only men, drivers, smokers and even middle-altitude residents did not change the effect.

**Conclusion:**

These findings confirmed high efficacy of pre-employment screening limiting access of workers with advanced conditions to work which later yielded low CVD incidence. In addition to conventional contraindications to work at high altitude, age and high BMI should be considered when a decision is made.

## Introduction

High altitude is a unique environment which has become popular for travel, but for decades has also been a terrain for mining and exploration in selected countries. High altitude exposes biological species to a combination of environmental factors including hypobaric hypoxia, progressing with altitude in almost linear fashion, low temperatures, corresponding to arctic conditions at an elevation of 3000–3500 meters above sea level and higher, and low air humidity [1–6]. Additionally, remoteness from densely populated areas should also be considered in this context. Short-term acclimatization to high altitude [6–8] along with a long-term adaptation through generations in those living at altitude has been widely studied [9–12], and much is now understood in the way humans and other biological species adapt to hypoxia at altitude [6, 13–15]. In brief, respiratory, cardiovascular and gas transporting systems are primarily compromised, which results in higher workload with the purpose to sustain sufficient tissue oxygenation [8, 16, 17]. In particular, sympathetic activation through hypoxia-activated peripheral chemoreceptors leads to increased heart rate and cardiac output; simultaneous hyperventilation improves oxygenation, but is associated with alkalemia resulting from lower CO_2_ partial pressure. This, in turn, causes enhanced urinary excretion of bicarbonate. With elapsing time, cardiac output may return to normal, but heartrate remains elevated [16].

Studies comparing lung function in healthy subjects at lowland and high altitude, including those in Central Asia and Americas have revealed clear differences reflecting acclimatization mechanisms [18, 18, 19]. Even more research efforts have been put to characterize cardiovascular adaptation and the risk at high altitude. Earlier studies in Central Asia and more recent studies in Ecuador have shown low cardiovascular risk in the highlanders [4–6, 20]. At present, epidemiological evidence on cardiovascular disease (CVD) at high altitude is conflicting [16], when lower ischemic heart disease burden in some studies [20] may be higher in the other [21]. Discrepancies pertain to arterial hypertension and diabetes, but mortality from CVD has been consistently lower at high altitude [16, 20].

As opposed to amateur travel to high altitude for sports or recreation, employment in various industries in the mountainous areas exposes workforce to both high-altitude environment and conventional occupational hazards, most pronounced in the high-altitude mining industry [22–25]. In addition to industrial aerosol, noise, vibration, shift work, chemicals and remote employment, workers have to acclimatize to hypobaric hypoxia, usually in an intermittent manner, because work shifts at the mines at altitude alternate with rest at low altitude, possibly inducing even more stress [24, 26–28]. Because there exist only few high-altitude mines around the world, research and evidence on the mechanisms of adaptation in these specific occupational groups still remain sparse, and many aspects of well-being at high altitude from the public health perspective remain poorly understood. A few published cohort observations in the high-altitude miner cohorts elucidated mechanisms of long-term acclimatization [24, 29], clinical aspects of baseline diseases during intermittent exposure [30], predictors for premature drop-out from work [31] and even the ways acute mountain sickness can be prevented [32].

However, the interplay of high-altitude environment, occupational hazards in the workplace, chronic medical conditions and diseases as well as lifestyle will eventually determine whether a subject can or cannot work at altitude. Therefore, the way how chronic diseases will evolve is pivotal to sustain healthy workforce for high-altitude mining. Cohort studies have demonstrated that selected individuals with a range of medical conditions will decompensate at high altitude, but some may benefit from working at altitude, such as patients with bronchial asthma even when occupational hazards are present in the workplace [30]. Nevertheless, conditions under which a worker will develop a chronic disease, such as a CVD, that will make him or her unfit to work, yet remain poorly understood and understudied. Because CVD, including ischemic heart disease, still remains the one associated with the largest burden and high mortality rates, we focused the current study on a group of cardiovascular conditions and hypothesized that occupational hazards, cigarette smoking, poor baseline cardiovascular health may predict the onset of such diseases making worker unfit to work. Hence, the aim of the current study was to define the incidence of CVD leading to unfitness to work as well as their predictors in a prospective observation of the cohort of high-altitude mining workers.

## Materials and methods

### Study design

This study was approved by the Committee on Bioethics of Kyrgyz State Medical Academy (approval #35/2008 dated 13.06.2008), and all subjects signed an informed written consent to participate once they were enrolled in the pre-placement medical screening. This is a prospective observational cohort study of the newly hired employees for a high-altitude gold mine in Kyrgyzstan. Detailed information on the cohort and the mine is presented in the first publication [32], when data on the probability and predictors of acute mountain sickness in this occupational group were analyzed after one full year of observation. Later, we also analyzed blood pressure change in the employees after full year of employment [33], but recently we also demonstrated how healthy worker effect developed in this cohort elucidating predictors of premature drop-out from work [31]. In general, this gold mine is one of the most elevated open-pit gold mines in the world, situated in Tien Shan Mountain Gorge in the South of Kyrgyzstan, almost 600 km away from its capital, Bishkek. Residential camp is located at an elevation of 3800 meters above sea level, mill at 4000 meters and the pit spreads to an elevation of 4500 meters.

### Participants

Subjects were consecutively recruited from January 1, 2009 to December 31, 2012. Study sample size was defined based on the time frame of the initial enrollment of participants into this fixed cohort: all prospective employees undergoing screening for pre-employment were considered potential cohort members, unless they did not meet inclusion criteria. Therefore, we screened all who underwent medical examination from 2009 till 2012.

Most workers reside at middle altitude in the villages mostly along the southern shore of Issykul lake (1600 meters above sea level), but a few also commute from Bishkek for their two weeks on-site shift with the subsequent two weeks of rest at home. Transportation to the mine with buses takes four hours from Issykul villages, when study participants rapidly ascend from 1600 to 3800 meters and proceed to their work shift next morning. Two weeks of shifts at the mine usually comprise 12 hours of work with 12 more hours of rest within one day, and when at the mine, workers move within the mine from the altitude 3800 to 4500 meters. Such pattern of exposure to high-altitude climate in the mine employees fits within “chronic intermittent hypoxia” pattern, exposing them to hypobaric hypoxia, cold climate, wind and dry air in addition to a conventional set of occupational hazards of the gold mining and extraction using cyanides.

### Pre-employment and annual screening

In Kyrgyzstan, medical clearance for work is obtained at pre-employment and annual screening, as required by the local legislation. All high-altitude employees must have chest X-ray, 12-lead electrocardiogram (ECG), blood cell count, blood biochemistry, urine test, audiometry and office spirometry with bronchodilation in addition to physical examination with a panel of doctors. This panel comprises internal diseases doctor, dermatologist, gynecologist, ENT, ophthalmologist, surgeon, psychiatrist, neurologist and a cardiologist. Any of these specialists may refer subjects to ancillary tests and examinations, such as stress-ECG, coronary angiography and other. We extracted data from the medical examination, whereas one of the authors worked as an internist in this panel. Among a variety of collected parameters, we used age, occupation, place of residence, smoking status, height, weight, blood pressure, heart rate (HR), SaO2 at the very first ascent to mine site, spirometric indices (defined below) and all diagnostic codes claimed by each doctor in a panel.

For this study, we collected all available tests data and medical records including recorded diagnoses from subjects undergoing pre-employment screening in 2009, 2010, 2011 and 2012. Exclusion criteria were: students undergoing examination for summer internships; all visitors to site; all those who worked for less than one year; and employees of Issykul marshalling yard and Bishkek offices (not at high altitude). Smoking status definition and lung function protocols were described in more detail before [32, 34]. Occupational group was ascertained later and data were matched with the human resources roster.

### CVD definition and follow-up

Once subjects were enrolled, an observation stared, an those with work duration less than one year were later excluded from the analysis. At every subsequent mandatory annual screening, which was equipollent to a pre-employment screening, a cardiologist in the panel screened workers to selected a high-risk group and decided whether they needed an advanced examination at a tertial level facility with stress-ECG, lipid profile test, cardiac ultrasonography and hypobaric chamber test and coronary angiography upon indications. Alternatively, subjects were referred to such examination if an event happened at site that could prompt a CVD, including fainting, chest pain, abrupt shortness of breath, dizziness, palpitations, etc. Once the diagnosis was ready, a panel then made decision if a person was now fit to work with this new diagnosis based on the legal list of contraindications. Therefore, the current analysis treats cardiovascular cases as those in which a diagnosis was confirmed at a tertial level facility, were non-fatal and who subsequently were claimed unfit to work. These diagnoses included all forms of coronary artery disease (CAD), such as cardiac death, myocardial infarction, angina, and clinically significant coronary atherosclerosis (ischemia with no signs); rheumatic disease with a cardiac defect; ascending aorta dilation; high-risk arterial hypertension; high-grade obesity with other signs (elevated cholesterol, etc.); all arrythmias; and congenital defects combined with other signs. Workers with a CVD who were claimed fit to work because of no formal contraindications, such as those with well-controlled conditions and low risk of deterioration were not treated as cases in this analysis. Furthermore, those who died, also from confirmed or non-confirmed cardiovascular event, were not cases in the current study.

### Statistical analysis

In this cohort observation, we firstly report the cumulative incidence of cases throughout the observation period. The second end-point in this presentation is the risk of acquiring unfitness to work due to a CVD. Data were tested for normality, and many variables were found non-normally distributed; therefore, we only used and reported non-parametric tests in this project. The frequency of the outcome of interest was reported for each binary variable in N and percent of the group. In the univariate comparisons of categorical variables, we tested the differences using non-parametric χ2 tests with contingency tables, and such variables were sex, place of residence (low altitude vs. middle altitude), daily smoking, occupational group (physically demanding vs. non-physically demanding job), as well as all baseline diagnosis from the medical record. Continuous variables were age, lung function (vital capacity (VC) as actual and percent predicted, forced VC (FVC) as actual and percent predicted and forced expiratory volume in one second (FEV_1_) as actual and percent predicted), cigarettes smoked per day for daily smokers, body mass index (BMI), systolic and diastolic blood pressure and HR at the annual screening, and SaO_2_ at the very first ascent to mine site. We tested each of these in those found unfit to work at follow-up with the alternative group.

We then applied Cox regression models to test whether each of these predictors was associated with the probability to obtain CVD diagnosis with unfitness to work in the univariate comparisons. All subjects who retained at work by January 2022 were censored in this analysis. Variables found significantly associated with the outcome were then included in a multivariate model, in which all predictors were adjusted for each other. In all regression models, we reported the hazard ratios (HRs) with their corresponding 95% confidence intervals (CI) for each predictor, including categorical and continuous ones. Once the analysis was completed, we further narrowed the analysis to males, then drivers, smokers and, finally, to middle-altitude residents and performed such stratification analysis also reporting HRs. All tests were completed in NCSS 2022 (Utah, USA), and p-values below 0.05 were considered significant.

## Results

Overall, 569 workers were included in the cohort and observed by January 2022 with the total 5190 person-years of observation. The median age of this predominantly male cohort (Table 1) at the time of entry was 34 (interquartile range (IQR) 28;40) years. As expected, cigarette daily smoking prevalence was high, and reached 53% in the group of vehicle operators (Table 1). Smokers reported to smoke 8 (5;10) cigarettes per day. Table 1 also shows that the groups of vehicle operators were almost completely made of mid-altitude residents, and those who were employed from lowland (the capital, Bishkek) were recruited to the alternative positions. The group in general demonstrated excellent lung function, blood pressure, HR and the overall cardiovascular health. Thus, only 8% of newly hired employees were obese, but 23% of participants already had some signs of acclimatization to high altitude, such as hemoglobin > 180 g/L, assuming some exposure in the past. Vehicle operators differed from other occupations not only with regard to residence, age, sex and smoking status, but had also higher VC, FVC, BMI and systolic BP.

**Table 1:** The summary of measured lung function and cardiovascular basic signs of the cohort overall and stratified by occupation.

Characteristics	All (n = 569)	Vehicle operators (n = 284)	Other occupations (n = 285)
*Demographics and smoking*			
Lowland residents, n (%)	88 (15)	7 (2)	81 (28)*
Age at entry, years, median (IQR)	34 (28;40)	36 (31;41)	31 (26;38.5)*
Males, N (%)	541 (95)	284 (100)	257 (90)*
Current cigarette smoking, n (%)	253 (44)	151 (53)	102 (36)*
*Physiological Variables*			
VC, % predicted, median (IQR)	103.1 (96.5;111.0)	105.6 (98.8;113.1)	101.9 (95.8;108.9)*
FVC, % predicted, median (IQR)	110 (103;119)	112 (104;120)	109 (102.5;116)*
FEV_1_, % predicted, median (IQR)	105.1 (96.7;113.1)	106.3 (96.6;115.5)	104.6 (96.7;111.9)
FEV_1_/FVC %, median (IQR)	80 (76;85)	80 (75;84)	80.9 (77;86)*
BMI, kg/m^2^, median (IQR)	24.2 (22;27.1)	24.8 (22.6;27.6)	23.6 (21.4;26.6)*
Obese, n (%)	46 (8)	26 (9)	20 (7)
BP systolic, mm Hg, median (IQR)	122 (120;130)	125 (120;130)	120 (115;130)*
BP diastolic, mm Hg, median (IQR)	80 (70;80)	80 (70;80)	80 (70;80)
Heart rate, beats per minute, median (IQR)	70 (66;78)	70 (64;80)	70 (66;78)
Hemoglobin, g/l, median (IQR)	170 (160;180)	171 (161;180)	170 (159;180)
SaO_2_ at first ascent, %, median (IQR)	89 (87;91)	89 (87;91)	89 (87;90)

IQR—interquartile range; FEV_1_—forced expiratory flow in one second; VC—vital capacity; FVC—forced VC; BMI—body mass index; BP—blood pressure; SaO_2_—blood oxygen saturation. Lowland is defined by residence at less than 1000 meters above sea level.

*—p<0.05

By the end of observation (median time of observation 119 (IQR 102; 139) months), when all workers were censored, 155 (27%) workers were found to have left the job. Of these, nine workers died, and the majority of those deceased (88%) at home. Reasons of death have been described in more detail earlier [31]. The cumulative incidence of CVD diagnoses with unfitness to work was as low as 4% or 4 per 1000 workers per year (23 cases). We found no difference in this incidence between drivers (N = 12 cases) and non-drivers (N = 11 cases). These 23 cases included 7 CAD cases; 3 cases of rheumatic disease with a cardiac defect; 1 case of ascending aorta dilation; 6 cases of high-risk arterial hypertension; 5 cases of arrythmias; and 1 case of congenital defects (Table 2).

**Table 2:** Cardiovascular diagnoses associated with unfitness to work in the cohort and their cardiovascular parameters at pre-employment.

	Age of unfitness	BMI	BPsys	BPdia	HR
CAD (N = 7)	52 (48;60)	25.8 (22.3;29.7)	120 (110;120)	80 (70;81.2)	66 (66;78)
Rheumatic disease (N = 3)	40 (31;49)	26.7 (19.9;26.9)	125 (125;130)	80 (65;80)	72 (66;75)
Aorta dilation (N = 1)	56	23.1	130	80	65
High-risk arterial hypertension (N = 6)	53 (38.5;57)	31 (29;33)	135 (129;154)	91.5 (80;96)	75 (67;86)
Arrhythmia (N = 5)	41 (39;46)	23.5 (21;28)	120 (115;135)	80 (72.5;82.5)	76 (69;85.5)
Congenital defect (N = 1)	39	29.1	130	80	80

CAD–coronary artery disease; BMI–body mass index; BP–blood pressure; HR–heart rate. Date shown as medians with the interquartile range if not one observation in the group

In the univariate comparisons, we tested sex, age, year of employment, place of residence, occupation, smoking, exhaled CO, all three lung function parameters, both as actual and per cent predicted, blood pressure, HR, hemoglobin, height, weight, BMI, SpO_2_ at the first ascent, as well as each of 27 most prevalent diagnoses identified at the pre-employment screening in their association with the outcome of interest. Sex, current smoking, exhaled CO, place of residence, occupation, HR, systolic BP, SpO_2_ at the first ascent were not found associated with the outcome. The strongest association was detected for age, BMI and year of employment in these univariate comparisons, whereas some, but still significant association was found for diastolic BP, FVC% predicted, arterial hypertension and sensorineural deafness at baseline.

When we tested significant predictors in the adjusted for each other model (Table 3), we found diastolic blood pressure, arterial hypertension and sensorineural deafness no more associated with the outcome. Age, lung function and BMI, however, were associated with a greater chance of having CVD diagnosed with unfitness to work in the follow-up, adjusted also for year of employment and baseline diagnoses. The effect ranged from HR 1.04 to 1.18, stronger for BMI. In order to rule out the effect modification and to further explore the effect size, we limited the analyses to males only and observed a similar pattern. In a stratified analysis in drivers, both age and lung function demonstrated greater HRs, but BMI lost its effect. In a subgroup of daily cigarette smokers, we noted a similar pattern with a stronger effect of BMI. Moreover, unlike the overall group and other subgroups analyses, regression analysis in smokers yielded a significant association of not only FVC, but also of VC and even FEV_1_, when each used as the only lung function parameter in the model with a pattern observed for FVC. Finally, the analysis limited to middle-altitude residents (lowlanders excluded) resembled the pattern of a test in drivers. This can be explained by the fact that most drivers were mid-altitude residents. In addition, the effect of age was the most consistent across all stratified analyses.

**Table 3:** Hazard ratios with their corresponding 95% confidence intervals of the cardiovascular diagnosis with unfitness to work for selected predictors.

Predictor	Overall	Males	Drivers	Smokers	Mid-altitude residents
Age	1.13 (1.06;1.22)	1.13 (1.05;1.21)	1.16 (1.02;1.31)	1.17 (1.02;1.34)	1.16 (1.06;1.27)
FVC% pred.	1.04 (1.00;1.08)	1.04 (1.00;1.08)	1.06 (1.01;1.11)	1.08 (1.02;1.15)	1.06 (1.02;1.11)
BMI	1.18 (1.04;1.34)	1.18 (1.04;1.34)	N/S	1.31 (1.05;1.63)	N/S
Year of employment (2009 vs the rest)	3.84 (1.58;9.35)	3.78 (1.55;9.22)	N/S	N/S	N/S

Note: All models are adjusted for each variable in the table, diastolic blood pressure, arterial hypertension and sensorineural deafness, which were all found non-significantly associated with the outcomes in the entire groups and in each subgroup. FVC–forced vital capacity; BMI–body mass index; N/S–non-significant.

## Discussion

This is the first cohort observation of mining workers at an altitude of 4000 meters above sea level, in which cardiovascular diagnoses leading to further unfitness to work were monitored for an average of 12 years. In all newly hired and generally healthy employees for a high-altitude mine in Kyrgyzstan, we assessed the overall incidence of these diagnoses and tested selected predictors for such diagnoses. We found that the cumulative incidence of diagnoses was very low. We then demonstrated that advancing age, greater BMI and FVC, independent of smoking, occupation, sex and baseline diagnoses could increase the risk of further unfitness to work due to CVD, and the effect persisted when the group was restricted to men, drivers, smokers and even mid-altitude residents. Because similar studies have never been published before, these findings are hard to compare to other settings, but these results should, nevertheless, serve the basis to integrate evidence into the current but outdated regulatory documentation on pre-employment and annual screening of high-altitude mining workers.

We planned the current study with the purpose to provide further evidence support to decide whether a worker is fit to work at high altitude and whether his/her current mild but chronic conditions, physiological variables and lifestyle would increase the risk of premature work discontinuation due to unfitness in future. Since patients with severe and apparently worsening conditions and diseases were initially not allowed to work and only the remaining healthy subjects were recruited, we authorized subjects with mild conditions to the go to the mine to see if these workers would still be at greater risk to obtain unfitness to work with a cardiovascular event. We found that none of the mild baseline conditions and diagnoses were associated with such outcome. This confirmed a traditional notation that such patients may be allowed to work.

The cumulative incidence of non-fatal cardiovascular diagnoses making a worker unfit to work was very low and did not exceed 4% or 4 per 1000 workers per year. Because CVD is a multifactorial condition with a complex interplay of genetic, environmental and lifestyle factors, defining the contribution of high altitude is challenging. Furthermore, high-altitude exposure in this case is combined with a traditional array of occupational exposures, including noise and dust in the workplace. Therefore, such combined effect in high-altitude mining workers has yet to be elucidated. We could not find prospective observational cohorts of high-altitude miners with the corresponding analysis of cardiovascular events in the literature. Nevertheless, a recent prospective study (observation only 6 months) of healthy soldiers at high altitude has identified a range of predictors for such event, and those included age, BMI and even systolic BP [35]. Of 2141 soldiers at high altitude in the training dataset, 177 developed ECG signs of ischemia, corresponding to an incidence of 166 cases per 1000 subjects per year. Additionally, they focused the study on hematological data and identified a set of five lab parameters most useful for prediction using machine learning. However, some cases of myocardial ischemia might have been misclassified because the diagnosis was verified with ECG only, but not with imaging tests.

As with the study in Chinese soldiers, data from other studies have a limited applicability for high-altitude mining workers, because subjects are only exposed to high altitude, but not a combination of high altitude with mining occupational hazards. There is no consensus in the results of these studies. Thus, when hospital admissions and mortality in the high-altitude locations of Ecuador were compared to low altitude, high altitude yielded protective effect [20], but when patients with heart failure were considered, high altitude increased the risk of adverse events [36]. Furthermore, the largest published cohort of workers exposed to high altitude during the high-altitude railroad construction [30] could not clarify the risks for workers with CAD, because the diagnosis was a contraindication to work and subjects were not allowed to ascend to the worksite. The latter study, nevertheless, demonstrated the clinical prognosis of patients with AH at high altitude, but failed to generate evidence on the incidence of new cases and hypothesize the association with such work. Of note, new cases of arrythmia were analyzed in this cohort of railroad construction workers, and no life-threatening arrythmias were found.

However, we now found that greater BMI and advancing age should gain more attention when a decision on fitness is made. There now exists a strong evidence that age is one of the strongest non-modifiable risk factors for CVD in both sexes in general population in the world literature [37–39], but no formulation on that exists in the legislative basis for fitness to work decisions, including Kyrgyzstan state regulations. Hence, a panel of doctors to make decision on fitness faces difficulties in formally recommend unfitness to work for workers of advancing age. Our study clearly demonstrated that age increased the risk of unfitness to work with ongoing employment; therefore, we propose to include risk estimation based also on age for all newly hired employees, and this will apply to high-altitude mining only. Furthermore, high BMI and obesity have been reported to increase the risk of adverse events in trekkers at high altitude [40], mostly related to acute mountain sickness, and similar evidence exists for high-altitude workers [30, 41]; however, most of these studied analyzed short-term outcomes. The current study produced additional evidence that elevated BMI and obesity may be associated with unfitness to work in the future in a uniquely exposed group of high-altitude workers.

The current study also demonstrated excellent lung function at pre-employment as a reflection of healthy worker effect in the prospective employees. This finding is consistent with other observations from high-altitude mines [42] and living terrains [18, 19, 43], when high-altitude residents exhibited greater volumes compared to their lowland counterparts. Furthermore, a study in the Chilean miners showed that FVC could increase with years of employment, but our studies at the mine confirmed that smoking and occupational hazards may significantly suppress or even reverse this effect [23, 34]. Annual spirometry is thus an essential test to verify FEV_1_ and FVC decline and should be performed in all workers at high altitude mines irrespective of their exposure to dust or explosion gases.

The strength of this analysis is the long-term observation of mining workers, which reveals the effects of not only high altitude itself, but a combination of hypoxia with traditional mining hazards. A few mines are located at similar and even higher altitudes around the Globe, but collecting data at the mines in a prospective observational study is very challenging, and the current observation is the first in its kind.

### Limitations

Firstly, the analysis was limited to an initially defined list of predictors, collected at the pre-employment screening, whereas other unmeasured factors, such as lipid profile, blood biochemical markers, and a detailed history of leisure physical activity might have attenuated the effect and were not considered. Secondly, we could not consider the natural evolution of smoking status, occupation and other predictors along the employment and could only analyze baseline data. Thirdly, we could only include deaths, although a few, in the analysis, because misclassification was likely, given that autopsy in selected cases was not done. We could not have access to death certificates to ascertain reasons of death to accurately classify cardiovascular deaths; therefore, we planned the study in non-fatal cardiovascular events only.

This study has distinct implications for high-altitude occupational medicine. First and foremost, existing legislation necessitating pre-employment medical examination which aims to identify medical contraindications to work is efficient and prevents large adverse effects at the mines. However, it is solely based on a nosological approach and may sound outdated, given that the overall risk attributed to a range of potential predictors is not calculated and not considered. Age, BMI, smoking and physical activity should also be treated as attributes of high risk, even if no direct evidence for the last two was not obtained in the current study. Preceding observations, albeit with a shorter observation, support this recommendation. There is apparently a need to transfer to a new approach to a decision whether subject is fit to work in high-altitude mining which will no more be based on a binary conclusion, but on a total risk calculation. Future studies should concentrate on modelling these trajectories and consider a wider range of potential predictors, including cardiovascular age, lipid profile, physical fitness, and other yet unknown attributes. Albeit access to data on the follow-up of mining workers is hampered, the studies of this kind will dramatically ease work for a panel doctor with a decision.

## Conclusion

In conclusion, our findings confirmed high efficacy of pre-employment screening in its current form, which limits access of workers with advanced conditions to work. In addition to traditional contraindications to work at high altitude, age, BMI, smoking and other known and suspected cardiovascular risk factors should be considered as part of a newer model of risk assessment, because they may also be associated with cardiovascular outcomes and the associated unfitness to work. These data can be in future obtained from high-quality observational studies with high-altitude miners, whereas the employers should put effort to improve data availability for analysis.

## Supporting information

S1 Table Working file with baseline information. (XLSX)
